# Gene targets of mouse miR-709: regulation of distinct pools

**DOI:** 10.1038/srep18958

**Published:** 2016-01-08

**Authors:** Sneha Surendran, Victoria N. Jideonwo, Chris Merchun, Miwon Ahn, John Murray, Jennifer Ryan, Kenneth W. Dunn, Janaiah Kota, Núria Morral

**Affiliations:** 1 Department of Medical and Molecular Genetics, Indiana University School of Medicine, Indianapolis, Indiana, United States of America; 2 Division of Nephrology, Department of Medicine, Indiana University School of Medicine, Indianapolis, Indiana, United States of America; 3 Department of Biochemistry and Molecular Biology, Indiana University School of Medicine, Indianapolis, Indiana, United States of America

## Abstract

MicroRNA (miRNA) are short non-coding RNA molecules that regulate multiple cellular processes, including development, cell differentiation, proliferation and death. Nevertheless, little is known on whether miRNA control the same gene networks in different tissues. miR-709 is an abundant miRNA expressed ubiquitously. Through transcriptome analysis, we have identified targets of miR-709 in hepatocytes. miR-709 represses genes implicated in cytoskeleton organization, extracellular matrix attachment, and fatty acid metabolism. Remarkably, none of the previously identified targets in non-hepatic tissues are silenced by miR-709 in hepatocytes, even though several of these genes are abundantly expressed in liver. In addition, miR-709 is upregulated in hepatocellular carcinoma, suggesting it participates in the genetic reprogramming that takes place during cell division, when cytoskeleton remodeling requires substantial changes in gene expression. In summary, the present study shows that miR-709 does not repress the same pool of genes in separate cell types. These results underscore the need for validating gene targets in every tissue a miRNA is expressed.

MicroRNAs (miRNAs) are a class of small (~19–23 nt) non-coding RNAs that are widely expressed in plants, animals, and some viruses. It has been estimated that the human genome encodes over 2,400 miRNAs^1^, which regulate about 60% of mammalian genes^2^. Mammalian miRNAs can repress their targets through either protein translation inhibition or transcript destabilization (the predominant mechanism)^3^,^4^. An mRNA can be targeted by numerous miRNAs, and a single miRNA can target multiple mRNAs, which allows miRNAs to regulate multiple gene networks^5^. It is now widely accepted that miRNAs have important roles in regulating complex processes such as development^6^, cell cycle^7^, and metabolism^8^. Nevertheless, their role as regulators of gene expression is paradoxical. On one side, many miRNAs are highly conserved (sometimes even between vertebrates and invertebrates), which suggests functional importance^9^. On the other, deletion of individual miRNA often does not result in any obvious defects, implying that miRNAs are dispensable^10^. The view that is emerging from these studies is that, unlike transcription factors, most miRNA are not master regulators of gene expression^11^. Instead, miRNAs are fine tuners of transcription, contributing to set the mean level of expression of a gene, and buffering variations in expression due to environmental changes^12^. Thus, miRNAs confer robustness to transcriptional programs during transition from one developmental stage to another or during cell differentiation processes^13^.

miR-709 is an abundant miRNA expressed in multiple mouse tissues, including brain, thymus, heart, lung, liver, spleen, kidney, adipose tissue, and testes^14^,^15^,^16^,^17^. miR-709 is embedded in intron 8 of the Regulatory Factor X1 (*Rfx1*) gene, a member of the winged-helix subfamily of helix-turn-helix transcription factors with activation as well as repression activity^18^. Like miR-709, *Rfx1* is ubiquitously expressed^19^. A few studies have underscored the role of miR-709 in response to cellular stress and/or cell proliferation processes. In a mouse model of injury to the peripheral nervous system (PNS), miR-709 was found upregulated and shown to bind to the mRNA of transcription factors Egr2, c-Jun, and Sox-2, key mediators of dedifferentiation and myelination/demyelination^20^. In mouse testis, miR-709 controls expression of Brother Of the Regulator of Imprinted Sites (BORIS)^14^. BORIS is an important regulator of DNA methylation and imprinting, and controls epigenetic reprogramming during differentiation of germ cells^21^. In adipocytes, miR-709 plays a role on differentiation by targeting glycogen synthase kinase 3β (GSK3β)^15^. Finally, miR-709 has been shown to inhibit Notch1-induced T cell acute lymphoblastic leukemia (T-ALL) by targeting the oncogene c-Myc, Akt and Ras-GRF1^22^.

Every tissue possesses a distinctive transcriptome and miRNA signature. miRNAs expressed in multiple tissues would be predicted to bind to and regulate the same genes in these tissues, as long as the mRNAs were part of the tissue’s transcriptome. Currently, it is not known whether miR-709, a ubiquitous miRNA, regulates the same genes in different tissues. Here we have used a comprehensive approach to identify liver targets of miR-709, with special emphasis on analysis of previously validated targets in non-hepatic tissues.

## Results and Discussion

### miR-709 is highly abundant in mouse liver

It has been reported that only the most abundant miRNAs suppress their target genes, and about 60% are not active^23^. To identify miRNAs expressed in liver, miRNA profiles were obtained. Based on signal intensity, mmu-miR-709 (miR-709) is expressed at high levels in this tissue, at approximately one-fourth of the most abundant miRNA, miR-122, and ~2-fold higher than let-7a (Supplementary Table 1). Computational analysis of predicted targets using miRanda^24^ and miRWalk^25^ suggested that miR-709 gene targets are associated with cytoskeleton functions.

### miR-709 induces transcriptional silencing of cytoskeleton genes

**Figure 1:**
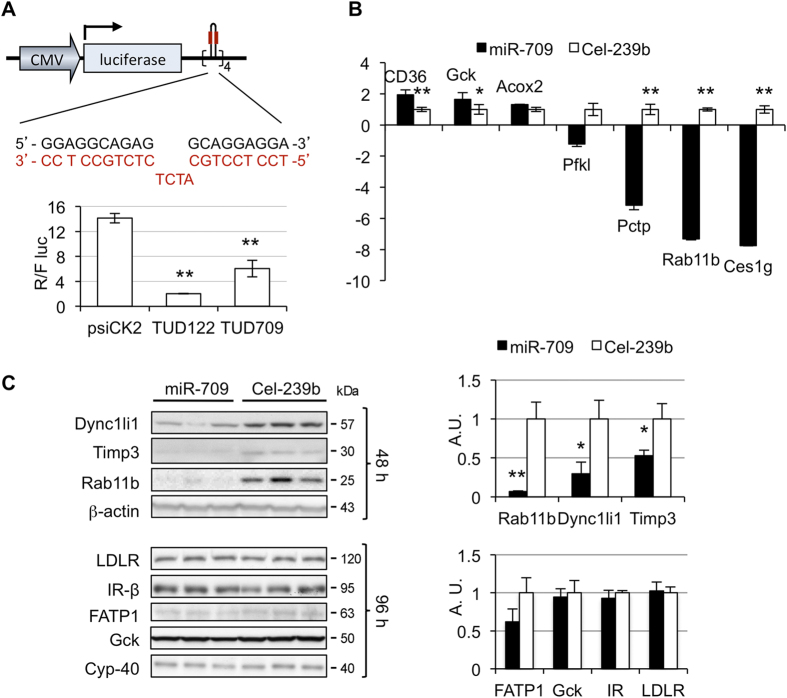
Targets of miR-709. (**A**) Primary hepatocytes were transfected with a tough decoy (TuD) containing 8 copies of the sequence complementary to the 3′ strand of miR-709 (shown in red; the sequence in black shows the mature 3′ strand of miR-709); TuD122: TuD for miR-122, used as positive control; psiCK2: psiCHECK2 plasmid without miRNA binding sites. Values represent mean ± SD (n = 3). The experiment was repeated in a separate hepatocyte isolation, with similar results. (**B**) Mouse primary hepatocytes were transfected with miR-709 or Cel-239b and harvested 24 hour later. Analysis of *CD36*, *Acox2*, *Rab11b*, *Pfkl*, *Pctp*, *Gck* and *Ces1g*, was performed by qRT-PCR. TATA binding protein (TBP) was used as normalizer gene. The fold change relative to Cel-239b for each gene is plotted. Values represent mean ± SD (n = 3–4). (**C**) Western blot analysis of proteins. Primary hepatocytes were transfected with miR-709 or Cel-239b and cells were harvested 48 hours or 96 hours later. Bands on blot were quantified by densitometry using ImageJ v1.48s, and results were normalized to control protein (Cyclophillin-40 or ß-actin). Values represent mean ± SD (n = 3). The experiment was repeated in a separate hepatocyte isolation, with similar results. *p < 0.05 and **p < 0.01, miR-709 vs Cel-239b.

Based on information available from the miRBase, the 3p strand of miR-709 is used for silencing (http://www.mirbase.org). We used luciferase reporter plasmids containing the complementary sequence to miR-709 (tough decoys), to confirm that in primary hepatocytes the 3p strand of miR-709 is used to repress its targets (Fig. 1A). The degree of luciferase repression was ~3-fold below the level observed with a tough decoy containing a target site for miR-122, the most abundant miRNA in liver, which is consistent with the amount of miR-709 relative to miR-122 (Supplementary Table 1). This indicates that miR-709 is expressed in hepatocytes and that the 3p strand is used for gene silencing.

It is widely accepted that mammalian miRNAs repress their targets mostly through mRNA destabilization rather than translation inhibition^4^,^26^. mRNA degradation accounts for 66 to 90% of miRNA-mediated regulation^27^. Therefore, we proceeded to identify miR-709 targets by transcriptome analysis. Mouse primary hepatocytes were transfected with miR-709 mimic or a control miRNA, Cel-239b, and harvested 24 hour later. This resulted in a 3.8-fold increase in cytoplasmic levels of miR-709 (Supplementary Fig. 1). Gene expression profiles were then generated using Affymetrix mRNA microarrays. Hierarchical cluster of the 100 genes with the lowest p-value indicated that all the samples within a treatment group cluster together (Supplementary Fig. 2). A total of 556 genes were downregulated and 848 were increased in miR-709-treated cells compared to Cel-239b (p < 0.01). Among genes downregulated, 36 were significantly decreased >2-fold in the miR-709 group compared to Cel-239b-treated control cells (Table 1), while only 4 were upregulated >2-fold (Table 2). Based on DAVID bioinformatics analysis^28^, the 36 genes are involved in lipid synthesis and transport (*Ces1*, *Pctp*, *Daglb*, *Cyp20a1*), cytoskeleton organization and endosomal recycling (*Rab11b*, *Dync1li1*, *Acta2*, *M6prbp1*, *Myo1d*, *Tagln*, *Cnn1*, *Sema6a*) and cell adhesion (*Timp3*, *Nid1*, *Thbs1*, *Krt19*, *Mpzl2*) (Table 1).

We then analyzed if there was any correlation between the extent of downregulation with target prediction using 3 databases: miRanda^24^, miRWalk^25^, and DIANAmT^29^. As many as 21 of the 36 genes downregulated >2-fold (58.3%) were predicted targets by all 3 databases, while the percentage dropped to 32.6% (170 out of 520) for genes downregulated <2-fold, and to 13.2% (112 out of 848) for upregulated genes. This suggests that there is a correlation between target prediction and fold-level downregulation, with the highest representation of predicted targets within the pool of genes that are downregulated above 2-fold. However, there was no correlation in the number of predicted miR-709 binding sites in the mRNA and the degree of downregulation: an average of 1.87 ± 1.04 binding sites for the 36 genes downregulated >2-fold, versus 1.79 ± 1.35 for a subgroup of 36 genes downregulated <2-fold (−1.23 to −1.25-fold), p = 0.82. Thus, factors different from the number of predicted binding sites in the mRNA are more likely to influence the extent of repression.

To validate the microarray results, several genes were analyzed by quantitative real time RT-PCR (qPCR), including *Cd36* (+1.8-fold), Acyl-Coenzyme Oxidase 2 (*Acox2*, +1.6-fold), Glucokinase (*Gck*, +1.5-fold), *Rab11b* (−4.1-fold), *Ces1g* (−3.2-fold), *Pctp* (−3.1-fold), Phosphofructokinase (*Pfkl*, −1.2-fold), and cytochrome P450, family 2, subfamily c, polypeptide 29 (*Cyp2c29*, +5.59-fold). Identical trends to those observed in the microarray analysis were observed (Fig. 1B, and Supplementary Fig. 3A). We then assessed whether mRNA downregulation resulted in changes in protein. Interestingly, no changes in protein levels were observed even after 4 days for genes that showed less than 2-fold difference in the microarray, such as *Gck* (+1.5-fold), *Slc27a1 (*or *Fatp1*, −1.9-fold), and Low-density Lipoprotein Receptor (*Ldlr*, −1.2-fold). Insulin Receptor (*Insr*, −1.1-fold, p = 0.42) was analyzed as a negative control (Fig. 1C, and Supplementary Fig. 3B). Protein changes did not correlate with target prediction in this group: *Slc27a1* (*Fatp1*) and *Gck* were predicted miR-709 targets, while *Ldlr* was not. *Insr* is a predicted target, and neither the mRNA or protein were changed by miR-709. Instead, genes whose mRNAs were downregulated multiple fold, such as *Rab11b* (−4.1-fold), *Dync1li1* (−3.4-fold), and *Timp3* (−3.2-fold), had significant decreases in protein levels (90%, 70% and 47% decrease, respectively; Fig. 1C). All three are predicted targets of miR-709.

**Figure 2:**
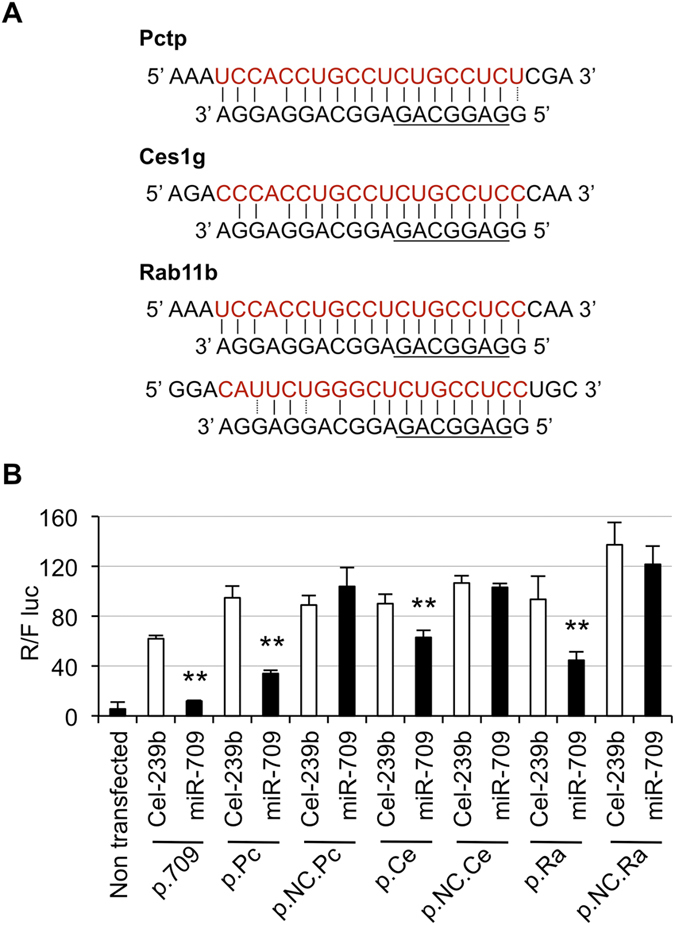
*Pctp*, *Ces1g*, and *Rab11b* are directly repressed by miR-709. (**A**) miR-709 binding sites in the 3′ UTR of Pctp, Ces1g and Rab11b mRNA. The binding sequence is shown in red, and the seed sequence is underlined. (**B**) Primary hepatocytes were transfected with miR-709 or Cel-239b, and plasmids containing the 3′ UTR of Pctp, Ces1g, or Rab11b. Twenty-four hours later, dual-luciferase assays were performed. Renilla luciferase activity was normalized to firefly luciferase expressed from the same plasmid. Values represent mean ± SD (n = 3). The experiment was repeated in a separate hepatocyte isolation, with similar results; p-values (**p < 0.01) are relative to cells treated with the same plasmid plus Cel-239b; p.709: plasmid containing the sequence perfectly complementary to miR-709-3p strand; p.Pc, p.Ce and p.Ra: plasmids containing a fragment of the 3′ UTR with miR-709 binding sites of Pctp, Ces1g and Rab11b, respectively; p.NC-Pc, p.NC-Ce and p.NC-Ra: plasmids with a fragment of the 3′ UTR without miR-709 binding sites.

To verify that the genes identified by microarray are direct targets of miR-709, we looked for predicted miR-709 binding sites on the 3′ UTR of three target genes using the miRanda database^24^. *Rab11b*, *Pctp* and *Ces1g* had 3, 4, and 1 binding sites, respectively. Plasmids containing a portion of the 3′ UTR of these genes, with or without predicted miR-709 binding sites (Fig. 2A), were generated and used in luciferase assays. As expected, lower levels of *Renilla* luciferase were observed with the constructs containing binding sites for miR-709 (p.Rab11b, p.Pctp and p.Ces1g), compared to constructs that had a fragment of the 3′ UTR without the miR-709 binding sequence (p.NC-Rab11b, p.NC-Pctp, and p.NC-Ces1g) (Fig. 2B). Furthermore, luciferase was lower only when miR-709 mimic –but not Cel-239b– was used. These data indicate that *Rab11b*, *Pctp*, and *Ces1g* are direct targets of miR-709, supporting the microarray results.

### miR-709 regulates a distinct set of genes in liver

**Figure 3:**
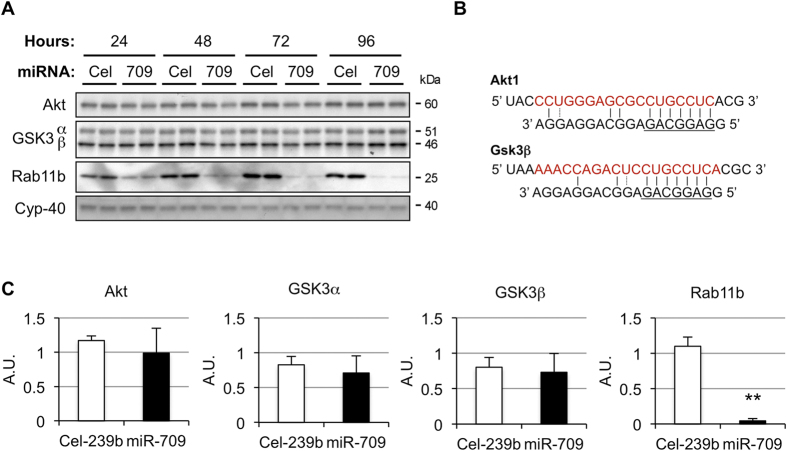
miR-709 does not repress Akt and GSK3β in primary hepatocytes. (**A**) Mouse primary hepatocytes were transfected with miR-709 or Cel-239b and harvested 24, 48, 72 and 96 hour later. Akt, GSK3β, Rab11b and Cyclophillin-40 were analyzed by Western blot analysis. (**B**) Previously validated miR-709 binding sites in *Akt1* and *Gsk3β*^15^,^22^. *Gsk3α* does not have binding sites for miR-709 (miRanda database^24^). (**C**) Bands from the 96 hour time point were quantified by densitometry using ImageJ v1.48s, and results were normalized to the loading control (Cyclophillin-40); values represent mean ± SD; data from 2 independent experiments were averaged (total of 6 replicates per group); **p < 0.01.

Given that miR-709 is a ubiquitously expressed miRNA, we then questioned whether it regulates the same genes in separate tissues. Eight genes have been previously shown to be validated targets of miR-709 in non-hepatic tissues (Table 3). Except for Gsk3β, which is repressed at the protein level only, all other genes are downregulated through a decrease in the amount of transcript^14^,^15^,^20^,^22^. Remarkably, none of these genes were considerably reduced by miR-709 in hepatocytes (Table 3). Only one was slightly increased (*Egr2*; +1.13), while a second was mildly decreased (*Akt1*; −1.18-fold), without leading to changes in protein (Fig. 3). Likewise, miR-709 had no impact on Gsk3β protein, a target in adipocytes (Fig. 3).

A miRNA that is expressed ubiquitously would be predicted to bind to and repress the same genes in different tissues, provided that the target genes were expressed in these tissues. Nevertheless, our data indicates that this is not necessarily the case. Indeed, half of the previously described miR-709 targets are abundantly expressed in liver [*Jun*, *Gsk3β*, *Myc*, *Akt1;* Table 3. As reference, albumin (*Alb*), fatty acid synthase (*Fasn*) and LDL receptor (*Ldlr*) have log_2_ signals of 13.5, 9.1 and 10.5, respectively]. Akt1 and Gsk3β, in particular, are important molecules in the insulin signaling pathway, regulating energy metabolism, glycogen synthesis, cell survival and cellular proliferation^30^. Neither one was significantly silenced by miR-709 in this tissue. Similarly, genes expressed at extremely low levels in liver, were not affected by miR-709 [*Egr2*, *Sox-2*, *Ctcfl*, *Ras-GRF1*; Table 3. Genes that are not normally expressed in hepatocytes such as gastric inhibitory polypeptide (*Gip*; expressed in intestinal cells) and glucagon (*Gcg*; pancreas-specific), have log_2_ probe signals of 4.8 and 3.4, respectively].

**Figure 4:**
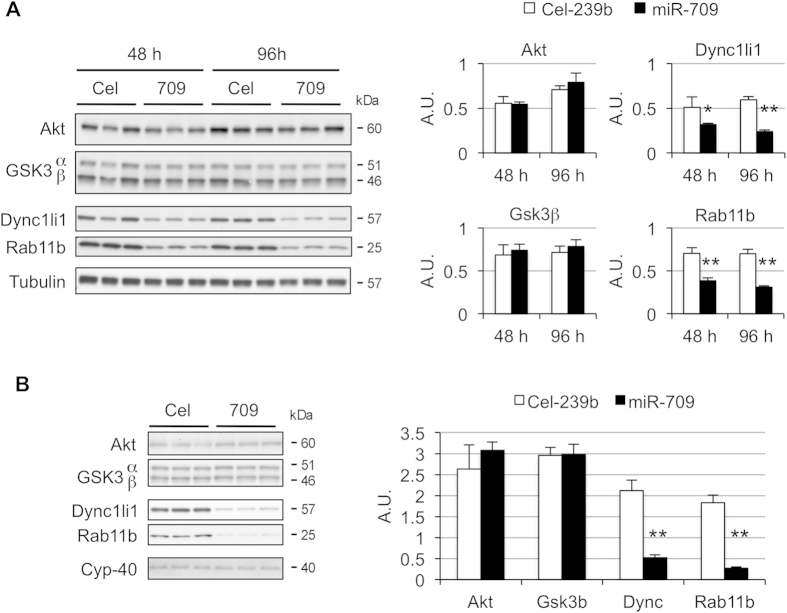
miR-709 does not repress Akt and GSK3β in 3T3-L1 fibroblasts and C2C12 myoblasts. (**A**) 3T3-L1 fibroblasts were cultured in 6-well plates and transfected with miR-709 or control Cel-239b. Cells were harvested after 48 and 96 hours. The densitometry analysis (ImageJ v1.48s) is shown on the right. Values represent mean ± SD (n = 3). (**B**) C2C12 myoblasts were transfected with miR-709 or control Cel-239b, and harvested after 48 hour. Akt, Gsk3, Dync1li1, and Rab11b were analyzed by Western blot. Values represent mean ± SD (n = 4); *p < 0.05, **p < 0.01.

To validate the newly identified miR-709 targets in other cell types, 3T3-L1 fibroblasts and C2C12 myoblasts were transfected with miR-709 mimic (Fig. 4). As observed in primary hepatocytes, *Rab11b* and *Dync1li1* were significantly downregulated in these cells. However, *Akt* and *Gsk3β* were not repressed, as had been observed in primary hepatocytes. miR-709 levels are lower in T cell acute lymphoblastic leukemia and during adipocyte differentiation, and this decrease is needed for the oncogenic and the differentiation process to occur^15^,^22^. Overall, the data suggest that some miR-709 gene targets may be regulated by this miRNA in multiple tissues (*Rab11b*, *Dync1li1*). However, other targets, including *Akt* and *Gsk3β*, may only be regulated by miR-709 in specific tissues and/or during oncogenic/developmental conditions. Thus, despite being a ubiquitous miRNA, miR-709 appears to control different gene networks in specific cellular processes and tissues, influencing distinct genetic programs.

### miR-709 is upregulated in hepatocellular carcinoma

**Figure 5:**
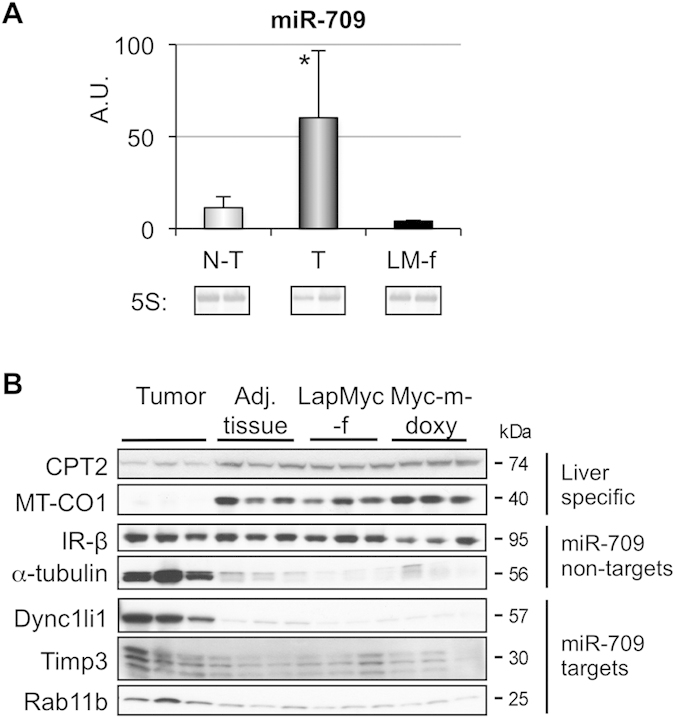
miR-709 expression in an animal model of hepatocellular carcinoma. (**A**) Mature miR-709 in LapMyc male (T: tumor and N-T: adjacent non-tumor tissue; n = 5) and LapMyc female (LM-f, negative control; n = 2) mouse livers were quantified by TaqMan assay. Values represent mean ± SD; *p < 0.05 between T and N-T groups. (**B**) Western blot analysis of MT-CO1, CPT2, IR-β, tubulin, and miR-709 targets (Rab11b, Dync1li1, Timp3) in tumor and control liver [adjacent non-tumor, LapMyc female and Myc male (doxycycline-treated) mice].

Multiple studies have provided evidence that most miRNAs exert only a mild repression on their targets^12^. This has prompted the notion that a general function of miRNAs is to tune and/or buffer expression of their targets, setting their mean level of expression and minimizing variance upon environmental changes. miR-709 is upregulated in response to cellular stress or tissue injury, and is involved in the genetic reprogramming that follows^14^,^15^,^20^. In peripheral nerve system (PNS) injury, miR-709 and miR-138 have opposing roles. miR-709 increases, repressing target gene expression, and miR-138 decreases, de-repressing expression^20^. The combined action of the miRNAs determines the end level of their targets: Egr2 (decreases in injury), Sox-2 (increases) and c-Jun (increases)^20^. Cytoskeleton reorganization is a distinctive feature of proliferative states such as hepatocellular carcinoma (HCC). In addition, a hallmark of HCC is the presence of genetic reprogramming towards a dedifferentiated state, during which liver-specific functions are shutdown^31^. We questioned whether miR-709 would be dysregulated in this process, as occurs in PNS injury^20^. Remarkably, levels of mature miR-709 were significantly upregulated (5.3-fold) in liver tumors (Fig. 5A), indicating that this miRNA is associated with the genetic reprogramming that takes place in HCC. Consistent with the dedifferentiated state^31^, proteins involved in liver-specific functions, such as carnitine palmitoyl transferase 2 (CPT2) and mitochondrially encoded cytochrome c oxidase I (MT-CO1) (enzymes of the fatty acid oxidation pathway) were downregulated in tumors (Fig. 5B). In contrast, the cytoskeleton protein α-tubulin, was robustly upregulated, as expected in cells that are actively dividing. Similarly, miR-709 targets that are involved in cytoskeleton function and attachment to the extracellular matrix, *Rab11b*, *Dync1li1* and *Timp3*, were upregulated in tumors relative to adjacent non-tumor liver and livers from control mice (Fig. 5B). These data suggest that the overall level of expression of these genes is influenced by the simultaneous action of multiple miRNA, some of which increase (like miR-709), while others decrease in HCC, as described in PNS injury^20^.

## Conclusions

In this study we have shown that the mRNAs of hundreds of genes are changed upon increasing miR-709 in hepatocytes. Nevertheless, the majority of the changes are lower than 2-fold, and do not necessarily lead to significant changes in protein levels. It is possible that repression of a number of genes occurs at the translational level instead of the mRNA, and additional genes from those identified through our microarray might be regulated by miR-709. In hepatocytes (and most probably in other cell types) miR-709 regulates structural and cell adhesion target genes, where it is likely to contribute to maintain the appropriate level of its targets during cell proliferation, thereby tuning/buffering gene expression. Increasing cytoplasmic levels of miR-709 has no impact on cell viability or proliferation (Supplementary Fig. 4). Despite its ubiquitous expression, miR-709 has distinctive targets during particular cellular processes, which underscores the complexity of gene regulation. Multiple factors can influence the dynamics of expression of any given gene, in addition to miRNAs. Transcription factors, mRNA tertiary structure, RNA conformation, and the presence of RNA binding proteins^13^, can influence the overall level of an mRNA. It is likely that one of these elements has a prominent role in determining the levels of a transcript in a specific tissue, while being less important in another. Thus, a miRNA may be essential in regulating an mRNA in one tissue, but not be critical in a different one. Overall, our data suggest that understanding the biological function of a miRNA may require carrying out studies in each tissue in which it is expressed.

## Materials and Methods

### Animals

All animal studies were in accordance with the National Institutes of Health guidelines and were approved by the Indiana University School of Medicine Institutional Animal Care and Use Committee. Four 12-week old, male C57BLKS/J mice were used to study miRNA expression profiles. Male C57BL/6J mice (24 to 30 g) were used for isolation of primary hepatocytes. Mice were purchased from The Jackson Laboratory (Bar Harbor, ME), and allowed to acclimate for at least a week before experimentation. A standard 12 h light/12 h dark cycle (7 AM/7 PM) was maintained throughout the experiments. Mice were fed rodent chow *ad libitum* and allowed free access of water.

The mouse model of hepatocellular carcinoma has been previously described^32^. Briefly, LapMyc mice express the *c-Myc* oncogene conditionally regulated by the Tet-Off system. The tetracycline-transactivator (tTA) protein is driven by the liver-specific promoter Liver Activator Protein (LAP), and the *c-Myc* gene (in the Y chromosome) has a tetracycline response element. In the absence of doxycycline, tTA can bind to the response element and cause c-Myc expression in male mice, inducing HCC. Expression of c-Myc was induced at 4 weeks of age and animals were euthanized at 14 weeks of age. Mice are maintained in the FVB strain. LapMyc female littermates, FVB wild type female and Myc-doxy male mice were used as negative controls.

### Primary hepatocyte isolation and cell culture

Primary hepatocytes were isolated from C57BL/6J mice using a two-step collagenase procedure followed by Percoll gradient centrifugation (to separate primary hepatocytes from non-parenchyma cells), as previously described^33^. Cell viability was assessed by trypan blue staining exclusion (>80% viability). Cells were seeded at a density of 4–6 × 10^5^ cells per well or 35-mm dish in DMEM supplemented with 10% (v/v) fetal bovine serum (FBS), 1% (v/v) penicillin/streptomycin (P/S), 3 nM insulin and 1 nM dexamethasone. Cells were incubated at 37 **°**C, 5% CO_2_ in a humidified incubator and allowed to attach for 4 hours. Media was then replaced with fresh media.

Hepa1c1c7 cells (American Type Culture Collection, Manassas, VA) were cultured in MEM-α supplemented with 10% FBS and 1% (v/v) penicillin/streptomycin (P/S). 3T3-L1 fibroblasts were cultured in DMEM supplemented with 10% bovine calf serum and 1% (v/v) P/S. C2C12 myoblasts were cultured in DMEM supplemented with 10% FBS and 1% (v/v) P/S. The 3T3-L1 fibroblast and C2C12 myoblast cell lines were kindly provided by Dr. Jeffrey Elmendorf.

### Plasmid cloning

Construct p.miR-709 was generated by cloning an oligonucleotide with a sequence perfectly complementary to the 3′ strand of miR-709 (based on the sequence published in TargetScan), downstream of the renilla luciferase gene in psiCHECK^TM^-2 (Promega, Madison, WI). Tough decoys (TuDs) binding miR-709 or miR-122 were generated by cloning 8 copies of the sequence complementary to the 3p strand of miR-709 or the 5p strand of miR-122, downstream from the luciferase gene in psiCHECK^TM^-2. The sequence inserted was synthesized with XhoI and NotI sites at the ends to facilitate cloning (GenScript, NJ, and Genewiz, NJ).

To confirm that Rab11b, Ces1g and Pctp are direct targets of miR-709, 150–300 base pairs of the 3′ UTR containing the putative binding sites [microrna.org^24^], were cloned in the NotI-XhoI site of psiCHECK^TM^-2. Total mRNA from mouse liver was used to generate the cDNA (High Capacity cDNA reverse transcription kit, Applied Biosystems, Grand Island, NY) and the corresponding portion of the 3′ UTR of Rab11b, Ces1g and Pctp containing the putative miR-709 binding sites was amplified by PCR using primers with restriction sites for NotI-XhoI (Supplementary Table 2). PCR products were cloned into psiCHECK^TM^-2, generating plasmids p.Rab11b, p.Ces1g, and p.Pctp. In addition, a portion of the 3′ UTR without miR-709 binding sites was cloned into psiCHECK^TM^-2 and used as negative controls (p.NC-Rab11b, p.NC-Ces1g, and p.NC-Pctp). Clones were sequenced prior to using them in luciferase assays.

### Cell transfection

Mouse primary hepatocytes or Hepa1c1c7 cells were co-transfected with plasmids (1.5 μg) and miR-709 or the control miRNA Cel-239b (1 μg) (Dharmacon, Lafayette, CO), or with these miRNAs alone (1 μg). Transfection was performed with Metafectene-Pro (Biontex, Munich, Germany), as described^33^. After overnight incubation, media was replaced with fresh media. For luciferase assays, cells were harvested 24 hours later and analyzed for luciferase activity using a Centro LB 960 Microplate Luminometer (Berthold Technologies, Oak Ridge, TN) and the dual-luciferase® reporter assay kit (Promega, Madison, WI). *Renilla* luciferase activity was normalized to firefly luciferase expressed from the same plasmid. 3T3-L1 fibroblasts and C2C12 myoblasts (4 × 10^5^ cell/well) were cultured in 6-well plates and transfected with 1.5 and 1 μg of miRNA, respectively. Cells were harvested after 48 or 96 hour.

### Microarray analysis

#### miRNA array

The long (>200 bp) and miRNA-enriched (<200 bp) RNA fractions were isolated using the mirVana miRNA isolation kit (Ambion, Austin, TX). The miRNA-enriched RNA fraction from normal, C57BLKS/J mouse liver was used to conduct miRNA chip analysis (LC Sciences, Houston, TX). The RNA from each sample was labeled and hybridized to each of four chips. Background was determined using a regression-based background mapping method. The regression was performed on 5% to 25% of the lowest intensity data points excluding blank spots. Raw data matrix was then subtracted from the background matrix. Normalization was carried out using a LOWESS (Locally-weighted Regression) method on the background-subtracted data. Transcripts were considered detectable if they met at least two conditions: signal intensity higher than 3xbackground standard deviation, and spot CV < 0.5. CV was calculated by (standard deviation)/(signal intensity). A transcript was listed as detectable only if the signals from at least 50% of the repeating probes were above detection level. Data adjustment included data filtering, log_2_ transformation, and gene centering and normalization. The data filtering removed miRNAs with (normalized) intensity values below a threshold value of 32 across all samples.

#### mRNA Affymetrix array

Four replicates for miR-709 and three replicates for Cel-239b were used. Total RNA was isolated from 1 × 10^6^ cells 24 hours post-transfection using RNeasy Midi kit (Qiagen) following the manufacturer’s protocol. The quality of RNA was determined by Agilent 600 Nanobioanalyzer. mRNA microarray hybridization was performed by the Center for Medical Genomics, at Indiana University School of Medicine. Affymetrix mouse gene 1.0 ST arrays were used to compare expression of about 28,850 genes using one chip per replicate. Data was analyzed using a 1-way Anova using log_2_-transformed signals. Principal component analysis (PCA) and hierarchical clustering of the top 100 genes was done. Data generated from this microarray has been deposited at the NCBI GEO repository under accession number GSE63875.

### qRT-PCR analysis

To analyze mRNA levels, qRT-PCR was carried out as described^34^ using the SYBR Green Qiagen One-Step reverse transcription-PCR kit (Qiagen, Valencia, CA) and the primer pairs described in Supplementary Table 2, in an ABI PRISM 7500 instrument (ABI, Foster City, CA). The TATA binding protein (*Tbp*) gene was used as loading control.

To quantify the level of mature miR-709, cDNA was generated from 10 ng of total RNA sample using the TaqMan MicroRNA Reverse Transcription Kit (Applied Biosystems, Foster City, CA). Quantitative PCR was performed with TaqMan MicroRNA Assays (Applied Biosystems) specific for miR-709 (P/N 001644) and sno-202 (P/N 001232).

### Western blotting

Primary hepatocytes and liver tissues from the hepatocellular carcinoma animal model were lysed in RIPA buffer (Thermo Scientific, Rockford, IL) containing protease and phosphatase inhibitors (Roche, Indianapolis, IN). Protein concentration was determined using the BCA kit from Pierce (Rockford, IL). Proteins (20–30 μg) were separated in 10% Tris-HCl SDS PAGE Criterion gel (Bio-Rad, Hercules, CA) and transferred to 0.2-μm PVDF membrane (Bio-Rad). Antibodies were used to detect α-tubulin (Thermo Scientific, Rockford, IL); Timp3, FATP1 (ACSVL5), β-actin, IR-β (Santa Cruz Biotechnology, Dallas, TX); Dync1li1 (GeneTex, San Antonio, TX); Cyclophillin-40, LDLR, MT-CO1 (Abcam, Cambridge, MA); Gck (Abgent, San Diego, CA); Rab11b, Akt and GSK3 (Cell Signaling, Danvers, MA); CPT2 antibody was a kind gift from Dr. Carina Prip-Buus (INSERM, U1016, Institut Cochin, Paris, France). HRP-conjugated secondary antibody was added and incubated at room temperature for 1 hour. Blots were developed with Pierce ECL kit (Thermo Scientific) and exposed to enhanced chemiluminescence (ECL) film (GE Healthcare, Piscataway, NJ).

### Northern blotting

miRNA-enriched (200 bp) RNA fractions were isolated from ~100 mg of liver using mirVana RNA isolation kit according to the manufacturer’s instructions (Ambion, Austin, TX). Four μg were separated on 15% TBE urea gels (Bio-Rad), transferred to Hybond-N membranes (GE Healthcare), and then UV-cross-linked using a Stratalinker 2400 (Stratagene, La Jolla, CA). 5S probe (100 pmol) was labeled with digoxigenin (DIG) using a 2^nd^ generation DIG oligonucleotide tailing kit (Roche, Indianapolis, IN). The probe was hybridized to membranes at 25 °C overnight in a hybridization oven after 2 hour of pre-hybridization at 60 °C. Three 2× SSC, 0.1% SDS washes were carried out for 10 min at room temperature followed by blocking and incubating with antibody against DIG. The signal was developed using CSPD (Roche) according to the manufacturer’s instructions.

### Statistical analysis

Data are presented as the arithmetic mean ± standard deviation (SD). Statistical differences between miR-709 and Cel-239b-treated groups were calculated using the unpaired two-tailed Student’s t-test. A *P* value of less than 0.05 was considered statistically significant. As indicated in the figure legends, experiments in primary hepatocytes were repeated in a separate hepatocyte isolation to confirm data.

## Additional Information

**How to cite this article**: Surendran, S. *et al*. Gene targets of mouse miR-709: regulation of distinct pools. *Sci. Rep*. **6**, 18958; doi: 10.1038/srep18958 (2016).

## Supplementary Material

Supplementary Information

## Figures and Tables

**Table 1:** Genes significantly downregulated >2-fold in miR-709-transfected primary hepatocytes (p < 0.01).

Gene symbol	Gene name	Cellular role	Fold change	p-value
*Tspan31*	Tetraspanin 31	Cell adhesion	−5.27	1.8E-06
*Rab11b*	Ras-related protein	Endosomal recycling	−4.18	6.5E-06
*Cyp20a1*	Cytochrome P450, Family 20, Subfamily A, Polypeptide 1	Lipid metabolism, detoxification	−3.76	1.2E-07
*Dync1li1*	Dynein, cytoplasmic 1, light intermediate chain 1	Cytoskeleton organization	−3.40	2.3E-07
*Bpnt1*	3'(2'), 5'-bisphosphate nucleotidase 1	Cytoplasmic, nucleotide hydrolysis	−3.28	9.3E-09
*Mmachc*	Methylmalonic aciduria (cobalamin deficiency) cblC type, with homocystinuria	Vitamin B transport	−3.27	1.5E-05
*Ces1*	Carboxylesterase 1	Lipid metabolism	−3.22	1.7E-06
*Timp3*	Tissue inhibitor of metalloproteinases 3	Cell adhesion	−3.20	4.6E-05
*Pctp*	Phosphatidylcholine transfer protein	Lipid metabolism	−3.19	6.2E-07
*Nid1*	Nidogen 1	Cell adhesion	−3.03	2.7E-06
*Slc35e1*	Solute carrier family 35, member E1	Unknown function	−2.95	3.7E-09
*Mare*	Alpha globin regulatory element containing gene	Unknown function	−2.75	7.0E-08
*D13Wsu177e (Nop16)*	DNA segment, Chr 13, Wayne State University 177, expressed	Cell proliferation	−2.71	1.7E-08
*Thbs1*	Thrombospondin 1	Cell adhesion	−2.67	2.1E-06
*Lrrc58*	Leucine rich repeat containing 58	Unknown function	−2.53	6.2E-07
*Acta2*	Actin, alpha 2, smooth muscle, aorta	Cytoskeleton organization	−2.52	1.4E-05
*M6prbp1*	Mannose-6-phosphate receptor binding protein 1	Endosomal recycling	−2.50	1.7E-06
*Atrn*	Attractin	Cell membrane (inflammatory response)	−2.46	4.7E-06
*Mpzl2*	Myelin protein zero-like 2	Cell adhesion	−2.41	4.1E-06
*Tagln*	Transgelin	Cytoskeleton organization	−2.36	6.6E-06
*Cnn1*	Calponin 1	Cytoskeleton organization	−2.36	4.0E-05
*Krt19*	Keratin 19	Cytoskeleton organization; Cell adhesion	−2.35	2.9E-03
*Myo1d*	Myosin ID	Cytoskeleton organization; Endosomal recycling	−2.31	1.5E-07
*Cyb5d2*	Cytochrome b5 domain containing 2	Lipid metabolism, detoxification	−2.29	1.1E-06
*Actc1*	Actin, alpha, cardiac muscle 1	Cytoskeleton organization	−2.29	1.3E-05
*Pfas*	Phosphoribosylformylglycinamidine synthase	Purine metabolism	−2.27	1.1E-08
*Ggcx*	Gamma-glutamyl carboxylase	Cytoplasmic, peptidyl-glutamic acid carboxylation	−2.20	7.5E-05
*Ccnyl1*	Cyclin Y-like 1	Cell proliferation	−2.17	1.0E-05
*Amt*	Aminomethyltransferase	Mitochondrion	−2.16	2.0E-04
*BC057893*	cDNA sequence BC057893	Unknown function	−2.12	3.3E-06
*Gpr155*	G protein-coupled receptor 155	Cell membrane, signaling	−2.05	1.7E-04
*Daglb*	Diacylglycerol lipase, beta	Lipid metabolism	−2.05	2.7E-06
*Sema6a*	Sema domain, transmembrane domain (TM), and cytoplasmic	Cytoskeleton organization	−2.04	9.4E-05
*Fnip2*	Folliculin interacting protein 2	Cytoplasmic, tumor suppressor	−2.01	3.9E-04
*Slc7a1*	Solute carrier family 7 (cationic amino acid transporter	Basic amino acid transport	−2.01	1.6E-06
*Mobkl2a*	MOB1, Mps One Binder kinase activator-like 2A	Cell proliferation	−2.00	2.3E-05

**Table 2:** Genes significantly upregulated >2-fold in miR-709-transfected primary hepatocytes (p < 0.01).

Gene symbol	Gene name	Cellular role	Fold change	p-value
*Cyp2c29*	cytochrome P450, family 2, subfamily c, polypeptide 29	Metabolism of foreign compounds	+5.59	5.8E-09
*A1cf*	APOBEC1 complementation factor	Apolipoprotein B metabolism	+2.05	2.7E-06
*Taf2*	TAF2 RNA polymerase II, TATA box binding protein (TBP)-a	Transcription initiation	+2.04	9.1E-07
*Hgd*	homogentisate 1, 2-dioxygenase	Catabolism of amino acids	+2.04	1.2E-05

**Table 3:** Previously validated miR-709 gene targets are not considerably modified in hepatocytes.

Gene symbol	Tissue	Ref.	Primary hepatocytes			
			Mean Cel*	Mean 709*	Fold change	p-value
*Egr2*	Sciatic nerve	20	4.65	4.83	+1.13	2.6E-02
*Sox-2*	Sciatic nerve	20	3.77	3.78	+1.01	9.2E-01
*Jun*	Sciatic nerve	20	8.84	8.77	−1.05	3.1E-01
*Ctcfl (BORIS)*	Testes	14	4.19	4.15	−1.03	6.8E-01
*Gsk3*β	Adipocytes	15	8.82	8.67	−1.11	7.5E-02
*Myc*	T cell acute lymphoblastic leukemia	22	10.02	9.99	−1.02	6.5E-01
*Ras-GRF1*	T cell acute lymphoblastic leukemia	22	4.01	3.93	−1.06	1.0E-01
*Akt1*	T cell acute lymphoblastic leukemia	22	9.41	9.17	−1.18	1.8E-03

^*^Log_2_ mean signal.

## References

[b1] Friedlander M. R. . Evidence for the biogenesis of more than 1,000 novel human microRNAs. Genome Biol 15, R57, doi: 10.1186/gb-2014-15-4-r57 (2014). 24708865 PMC4054668

[b2] Friedman R. C., Farh K. K., Burge C. B. & Bartel D. P. Most mammalian mRNAs are conserved targets of microRNAs. Genome Res 19, 92–105, doi: 10.1101/gr.082701.108 (2009). 18955434 PMC2612969

[b3] Huntzinger E. & Izaurralde E. Gene silencing by microRNAs: contributions of translational repression and mRNA decay. Nat Rev Genet 12, 99–110, doi: 10.1038/nrg2936 (2011). 21245828

[b4] Eichhorn S. W. . mRNA Destabilization Is the Dominant Effect of Mammalian MicroRNAs by the Time Substantial Repression Ensues. Mol Cell 56, 104–115, doi: 10.1016/j.molcel.2014.08.028 (2014). 25263593 PMC4292926

[b5] Jung H. J. & Suh Y. MicroRNA in Aging: From Discovery to Biology. Current genomics 13, 548–557, doi: 10.2174/138920212803251436 (2012). 23633914 PMC3468887

[b6] Lagos-Quintana M. . Identification of tissue-specific microRNAs from mouse. Curr Biol 12, 735–739 (2002). 12007417 10.1016/s0960-9822(02)00809-6

[b7] Di Leva G., Garofalo M. & Croce C. M. MicroRNAs in cancer. Annual review of pathology 9, 287–314, doi: 10.1146/annurev-pathol-012513-104715 (2014). PMC4009396 24079833

[b8] Lorenzen J., Kumarswamy R., Dangwal S. & Thum T. MicroRNAs in diabetes and diabetes-associated complications. RNA Biol 9, 820–827, doi: 10.4161/rna.20162 (2012). 22664916

[b9] Niwa R. & Slack F. J. The evolution of animal microRNA function. Curr Opin Genet Dev 17, 145–150, doi: 10.1016/j.gde.2007.02.004 (2007). 17317150

[b10] Miska E. A. . Most Caenorhabditis elegans microRNAs are individually not essential for development or viability. PLoS genetics 3, e215, doi: 10.1371/journal.pgen.0030215 (2007). 18085825 PMC2134938

[b11] Vidigal J. A. & Ventura A. The biological functions of miRNAs: lessons from *in vivo* studies. Trends Cell Biol, doi: 10.1016/j.tcb.2014.11.004 (2014). PMC4344861 25484347

[b12] Wu C. I., Shen Y. & Tang T. Evolution under canalization and the dual roles of microRNAs: a hypothesis. Genome Res 19, 734–743, doi: 10.1101/gr.084640.108 (2009). 19411598 PMC3647535

[b13] Ebert M. S. & Sharp P. A. Roles for microRNAs in conferring robustness to biological processes. Cell 149, 515–524, doi: 10.1016/j.cell.2012.04.005 (2012). 22541426 PMC3351105

[b14] Tamminga J., Kathiria P., Koturbash I. & Kovalchuk O. DNA damage-induced upregulation of miR-709 in the germline downregulates BORIS to counteract aberrant DNA hypomethylation. Cell Cycle 7, 3731–3736 (2008). 19029807 10.4161/cc.7.23.7186

[b15] Chen H. . miR-709 inhibits 3T3-L1 cell differentiation by targeting GSK3beta of Wnt/beta-catenin signaling. Cellular signalling 26, 2583–2589, doi: 10.1016/j.cellsig.2014.07.017 (2014). 25038456

[b16] Tang X. . A simple array platform for microRNA analysis and its application in mouse tissues. RNA 13, 1803–1822, doi: 10.1261/rna.498607 (2007). 17675362 PMC1986807

[b17] Jacobs M. E., Jeffers L. A., Welch A. K., Wingo C. S. & Cain B. D. MicroRNA regulation of endothelin-1 mRNA in renal collecting duct cells. Life sciences 118, 195–199, doi: 10.1016/j.lfs.2014.03.003 (2014). 24632479 PMC4163143

[b18] Gajiwala K. S. . Structure of the winged-helix protein hRFX1 reveals a new mode of DNA binding. Nature 403, 916–921, doi: 10.1038/35002634 (2000). 10706293

[b19] Lubelsky Y., Reuven N. & Shaul Y. Autorepression of rfx1 gene expression: functional conservation from yeast to humans in response to DNA replication arrest. Mol Cell Biol 25, 10665–10673, doi: 10.1128/MCB.25.23.10665-10673.2005 (2005). 16287876 PMC1291218

[b20] Adilakshmi T., Sudol I. & Tapinos N. Combinatorial action of miRNAs regulates transcriptional and post-transcriptional gene silencing following *in vivo* PNS injury. PloS one 7, e39674, doi: 10.1371/journal.pone.0039674 (2012). 22792185 PMC3391190

[b21] Loukinov D. I. . BORIS, a novel male germ-line-specific protein associated with epigenetic reprogramming events, shares the same 11-zinc-finger domain with CTCF, the insulator protein involved in reading imprinting marks in the soma. Proc Natl Acad Sci USA 99, 6806–6811, doi: 10.1073/pnas.092123699 (2002). 12011441 PMC124484

[b22] Li X., Sanda T., Look A. T., Novina C. D. & von Boehmer H. Repression of tumor suppressor miR-451 is essential for NOTCH1-induced oncogenesis in T-ALL. J Exp Med 208, 663–675, doi: 10.1084/jem.20102384 (2011). 21464222 PMC3135352

[b23] Mullokandov G. . High-throughput assessment of microRNA activity and function using microRNA sensor and decoy libraries. Nature methods 9, 840–846, doi: 10.1038/nmeth.2078 (2012). 22751203 PMC3518396

[b24] Betel D., Wilson M., Gabow A., Marks D. S. & Sander C. The microRNA.org resource: targets and expression. Nucleic Acids Res 36, D149–153, doi: 10.1093/nar/gkm995 (2008). 18158296 PMC2238905

[b25] Dweep H., Sticht C., Pandey P. & Gretz N. miRWalk--database: prediction of possible miRNA binding sites by "walking" the genes of three genomes. Journal of biomedical informatics 44, 839–847, doi: 10.1016/j.jbi.2011.05.002 (2011). 21605702

[b26] Guo H., Ingolia N. T., Weissman J. S. & Bartel D. P. Mammalian microRNAs predominantly act to decrease target mRNA levels. Nature 466, 835–840, doi: 10.1038/nature09267 (2010). 20703300 PMC2990499

[b27] Izaurralde E. Breakers and blockers-miRNAs at work. Science 349, 380–382, doi: 10.1126/science.1260969 (2015). 26206919

[b28] Huang da W., Sherman B. T. & Lempicki R. A. Systematic and integrative analysis of large gene lists using DAVID bioinformatics resources. Nat Protoc 4, 44–57, doi: nprot.2008.211 [pii]10.1038/nprot.2008.211 (2009). 19131956 10.1038/nprot.2008.211

[b29] Paraskevopoulou M. D. . DIANA-microT web server v5.0: service integration into miRNA functional analysis workflows. Nucleic Acids Res 41, W169–173, doi: 10.1093/nar/gkt393 (2013). 23680784 PMC3692048

[b30] Schultze S. M., Jensen J., Hemmings B. A., Tschopp O. & Niessen M. Promiscuous affairs of PKB/AKT isoforms in metabolism. Arch Physiol Biochem 117, 70–77, doi: 10.3109/13813455.2010.539236 (2011). 21214427

[b31] Klochendler A. . A transgenic mouse marking live replicating cells reveals *in vivo* transcriptional program of proliferation. Dev Cell 23, 681–690, doi: 10.1016/j.devcel.2012.08.009 (2012). 23000141

[b32] Beer S. . Developmental context determines latency of MYC-induced tumorigenesis. PLoS Biol 2, e332, doi: 10.1371/journal.pbio.0020332 (2004). 15455033 PMC519000

[b33] Park J. S., Surendran S., Kamendulis L. M. & Morral N. Comparative nucleic acid transfection efficacy in primary hepatocytes for gene silencing and functional studies. BMC Res Notes 4, 8, doi: 1756-0500-4-8 [pii]10.1186/1756-0500-4-8 (2011). 21244687 10.1186/1756-0500-4-8 PMC3033823

[b34] Witting S. R., Brown M., Saxena R., Nabinger S. & Morral N. Helper-dependent Adenovirus-mediated Short Hairpin RNA Expression in the Liver Activates the Interferon Response. J Biol Chem 283, 2120–2128 (2008). 18025090 10.1074/jbc.M704178200

