# Supplementary material for: Gene targets of mouse miR-709: regulation of distinct pools
Source: Sci Rep. 2016 Jan 8;6:18958. doi: 10.1038/srep18958 (PMC4705522; doi:10.1038/srep18958)
Supplement: Supplementary Information [file srep18958-s1.pdf]

Gene targets of mouse miR-709: regulation of distinct pools

Sneha Surendran[1], Victoria Jideonwo[1], Chris Merchun[1], Miwon Ahn[1], John Murray[1], Jennifer Ryan[2], Kenneth W. Dunn[2], Janaiah Kota[1] & Núria Morral[1,3*]

From the [1]Department of Medical and Molecular Genetics, Indiana University School of Medicine, Indianapolis, Indiana, United States of America; [2]Division of Nephrology, Department of Medicine, Indiana University School of Medicine, Indianapolis, Indiana, United States of America; [3]Department of Biochemistry and Molecular Biology, Indiana University School of Medicine, Indianapolis, Indiana, United States of America

*Corresponding author:

E-mail: nmorralc@iu.edu

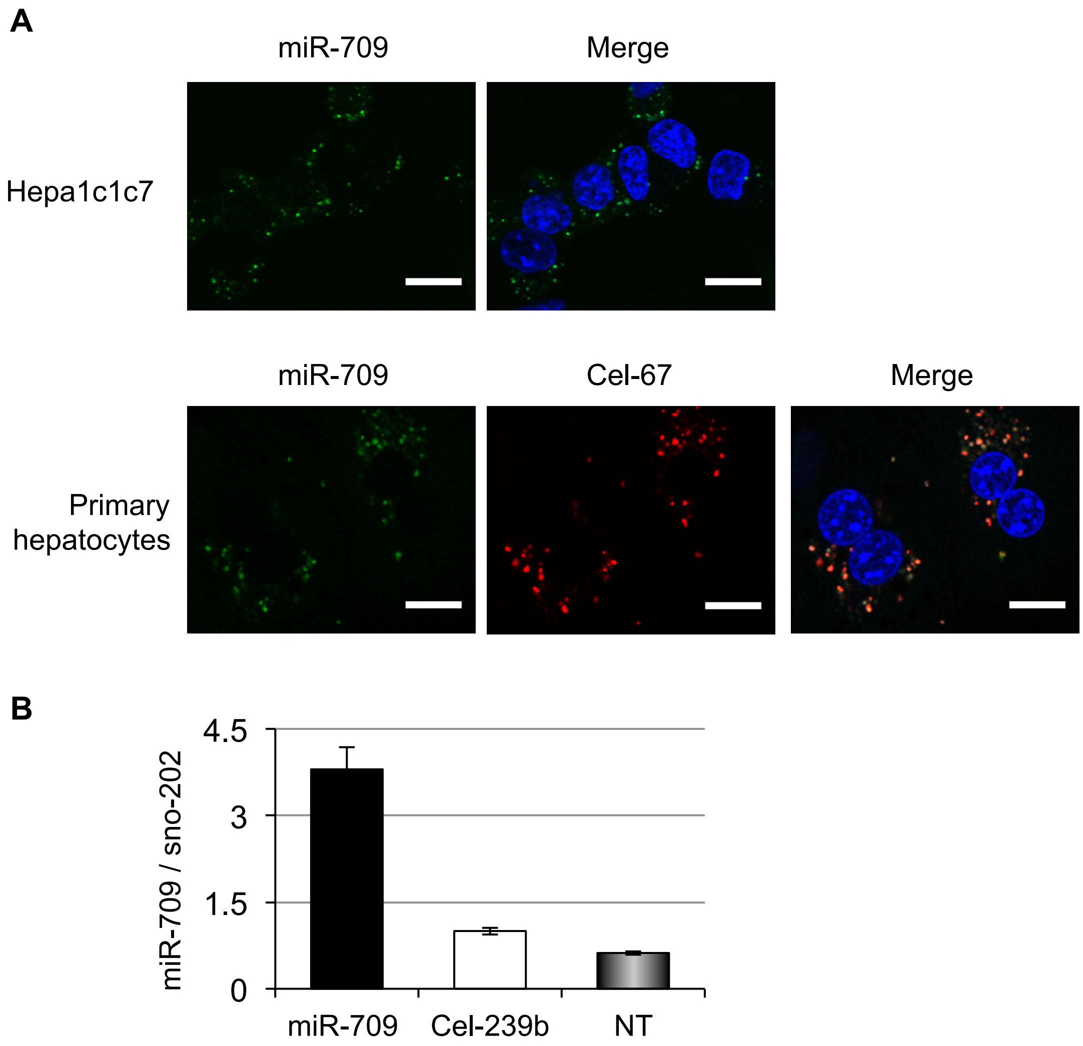

Sup. Fig. 1. Transfection with mimics increases cytoplasmic levels of miR-709. (A) Cytoplasmic localization of fluorescently labeled miR-709 mimic in mouse primary hepatocytes and Hepa1c1c cells. Primary hepatocytes (6x10^5) and Hepa1c1c (4x10^5) cells were plated on 35-mm glass bottom collagen coated dishes (MatTek, Ashland, MA). Next day, cells were transfected with 0.5 µg of 3'-FAM-labeled miR-709 only (Dharmacon, Pittsburgh, PA) (top, Hepa1c1c cells) or with 0.5 µg of Cel-67 Dy547-labeled control miRNA (bottom, primary hepatocytes). Twenty-four hours later, cells were imaged by confocal fluorescence microscopy on an inverted microscope fitted with an FV1000 MPE using a 60x1.2 NA water immersion objective lens (Olympus, Center Valley, PA). Nuclei were stained with Hoechst 33342 (NucBlue® Live ReadyProbes® Reagent; Molecular Probes, Grand Island, NY). Imaging was performed sequentially with excitation provided by solid state lasers at 405 and 559 nm and an Argon-ion laser at 488 nm; emission was collected with spectral filtration from 425-475 nm, 500-545 nm or filter based from 575-675 nm, respectively. Confocal slices were taken at 0.49 µm intervals with voxel dimensions of 0.265 x 0.265 µm. All post-acquisition analysis was performed in ImageJ v1.48p and figure generation was performed in Photoshop (Adobe, San Jose, CA). Scale bar, 15 µm. **(B)** Primary hepatocytes were transfected with 1 µg of unlabeled miR-709 mimic. Twenty-four hours later, quantification was performed by TaqMan assay. miR-709 levels were 3.8-fold higher than cells treated with Cel-239b. Data represent mean ± SD (n=2).

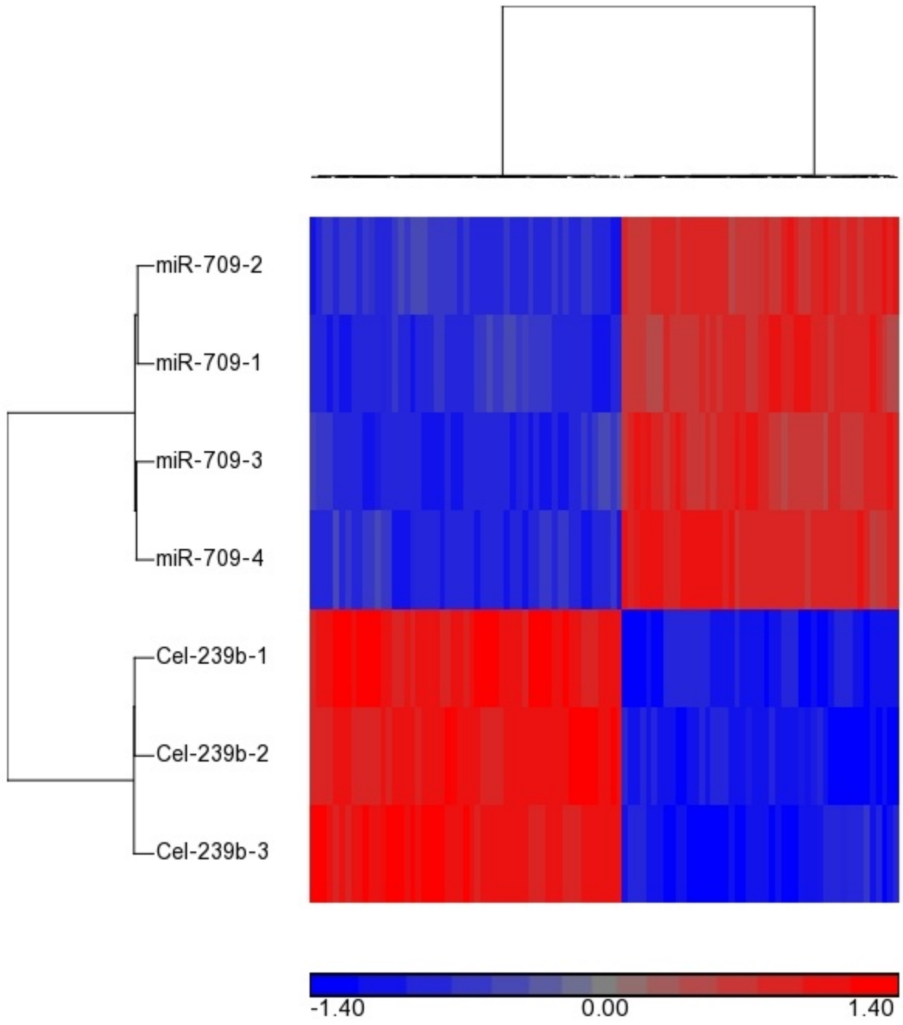

Sup. Fig. 2. Hierarchical cluster of top 100 genes. The 100 genes with the lowest p-value from ANOVA were selected for clustering. The Euclidean distance was used to measure the distance between samples, and Pearson's dissimilarity was used for genes. Cluster similarity was analyzed by the average linkage method. Numbers 1, 2, 3, 4 represent replicates of each miRNA treatment.

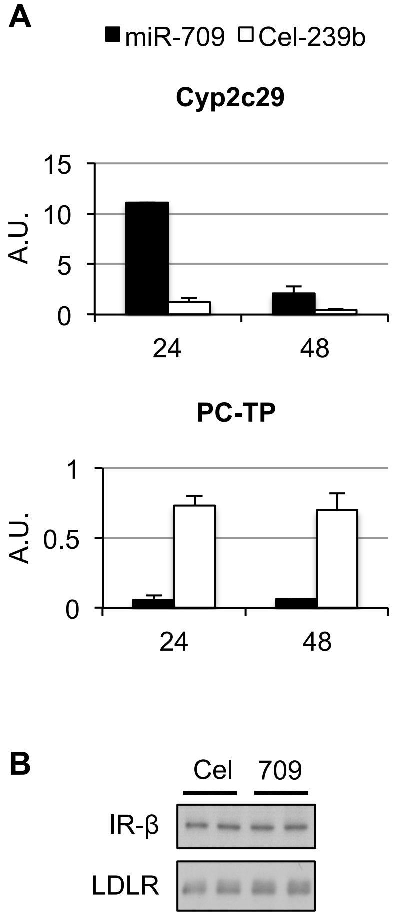

Sup. Fig. 3. Analysis of IRβ, LDLR, and Cyp2c2 at early time points post-transfection. Primary hepatocytes ($4x10^5$) were plated on 6-well plates and transfected with 1 μg of miR-709 or Cel-239b (n=2). **(A)** Cells were harvested 24 or 48 hours after transfection and total RNA was isolated. Quantification was performed by qRT-PCR. Data represents mean ± SD (n=2). At 24 hours, Cyp2c29 is increased 8.7-fold above Cel-239b, confirming the microarray data. Levels of miR-709 target PC-TP, are decreased. **(B)** Cells were harvested after 24 h and IRβ and LDLR protein was quantified by Western blot. No difference between Cel-239b and miR-709 was observed.

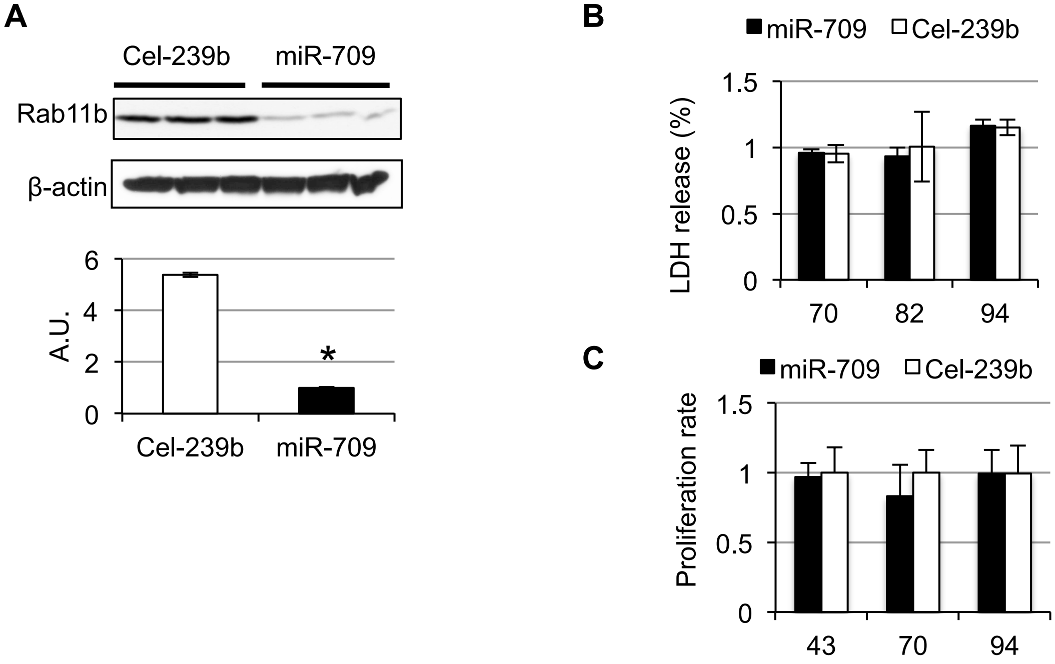

Sup. Fig. 4. MiR-709 does not influence cell viability and proliferation in mouse hepatoma Hepa1c1c cells. (A) Hepa1c1c cells were plated on 6-well plates at $4x10^5$ cells/well and transfected with 1 μg of miR-709 or Cel-239b (n=3). Media was replaced 24 hours post-transfection and cells were harvested 72 hours later. Rab11b protein expression was analyzed by Western blot. Bands on blot were quantified by densitometry and results were normalized to β-actin. Values represent mean ± SD. *$p<0.01$ relative to Cel-239b. **(B)** Cell viability based on LDH assay (Clontech, Mountain View, CA). Cells were treated as in (A), and medium was collected for LDH measurement. Values represent mean ± SD (n=6). Data at 70, 82 and 94 hours after transfection are plotted relative to 43 hours after transfection (when effects on protein levels are expected to be apparent). No changes in viability were observed between the two groups. The experiment was repeated, and similar results were obtained. **(C)** Cell proliferation assay, based on dehydrogenase activity (CCK-8, Dojindo Molecular Technologies, Rockville, MD). Cells ($1x10^5$) were plated in 12-well plates and transfected with 0.6 μg of miR-709 or Cel-239b. Media was replaced 24 hours later. Cell proliferation was determined at 43, 70 and 94 hours post-transfection. At each time point, data represent cell proliferation relative to the control Cel-239b. Values represent mean ± SD (n=4).

Supplementary Table 1. miRNAs expressed in liver of normal C57BLKS/J mice.

miRNA	Mean signal	S.D.
mmu-miR-122	50,406	15,046
mmu-miR-709	14,482	2,168
mmu-let-7a	6,797	1,504
mmu-let-7f	6,096	1,436
mmu-let-7c	6,086	811
mmu-let-7d	4,780	623
mmu-let-7b	4,103	369
mmu-miR-466f-3p	2,614	742
mmu-miR-26a	2,346	288
mmu-miR-467b*	1,842	499
mmu-let-7g	1,520	351
mmu-miR-192	1,463	227
mmu-miR-689	1,159	617
mmu-miR-23b	838	242
mmu-miR-466i	782	240
mmu-miR-720	778	455
mmu-miR-467f	774	253
mmu-miR-467a*	718	242
mmu-miR-466g	554	154
mmu-miR-574-5p	487	72
mmu-miR-669f	463	161
mmu-miR-191	456	170
mmu-miR-483	451	91
mmu-miR-705	378	132
mmu-let-7i	370	107
mmu-miR-690	369	48
mmu-let-7e	369	169
mmu-miR-21	355	355
mmu-miR-805	352	131
mmu-miR-24	350	125
mmu-miR-1895	343	66
mmu-miR-22	308	30
mmu-miR-762	301	107
mmu-miR-126-3p	293	120
mmu-miR-26b	283	245
mmu-miR-1224	262	72
mmu-miR-23a	258	52
mmu-miR-16	235	74
mmu-miR-125b-5p	231	80
mmu-miR-103	222	57
mmu-miR-1187	210	39
mmu-miR-194	209	47
mmu-miR-455	205	66

mmu-miR-107	197	45
mmu-miR-320	196	66
mmu-miR-29a	190	119
mmu-miR-574-3p	190	46
mmu-miR-214	175	74
mmu-miR-145	164	64
mmu-miR-31	157	59
mmu-miR-30c	155	49
mmu-miR-378	153	34
mmu-miR-1196	144	65
mmu-miR-27b	140	46
mmu-miR-151-5p	139	50
mmu-miR-30d	117	16
mmu-miR-92a	110	29
mmu-miR-423-5p	108	32
mmu-miR-361	105	34
mmu-miR-1892	91	35
mmu-miR-185	90	27
mmu-miR-568	84	47
mmu-miR-143	83	22
mmu-miR-1897-5p	80	18
mmu-miR-148a	69	71
mmu-miR-681	68	63
mmu-miR-669c	64	36
mmu-miR-489	64	64
mmu-miR-222	64	19
mmu-miR-25	58	10
mmu-miR-15b	57	28
mmu-miR-30a	56	13
mmu-miR-181a	52	9
mmu-miR-188-5p	49	9
mmu-miR-140*	49	22
mmu-miR-27a	42	13
mmu-miR-485*	42	7
mmu-miR-221	40	13
mmu-miR-680	39	30
mmu-miR-92b	37	4
mmu-miR-290-5p	37	23
mmu-miR-30b	36	17
mmu-miR-369-5p	35	53
mmu-miR-101b	34	12
mmu-miR-146a	33	16
mmu-miR-199a-3p	33	4
mmu-miR-20a	33	8
mmu-miR-99b	32	12
mmu-miR-674	32	11
mmu-miR-193	32	11

S.D., Standard Deviation

Supplementary Table 2. Oligonucleotides used to generate luciferase constructs, in Northern blots and primers used for qRT-PCR (5' → 3').

**Oligonucleotides**	
**Pctp**	TATGCACTCGAGCATCTGGATTTTTCCTTTCCC<br>ATCAGGCGGCCGCGGTGGTACACGCCTTTAATC
**NC- Pctp**	TATGCACTCGAGTCAGGCTTCAAAGATGGCTTG<br>AATCAGGCGGCCGCCTGAGTTCGAGGCTAACCTG
**Ces1g**	TATGCACTCGAGTCAATCGTCTGACACCAGTG<br>AATCAGGCGGCCGCTTCTAAGGGAATGTATTTGTGAT
**NC-Ces1g**	TATGCACTCGAGGAGCCAAGGAAACAGCAGAG<br>ATCAGGCGGCCGCTCCAGGAAAGCCAGGACTAC
**Rab11b**	ATGCACTCGAGCAGGGTTTCTCTGTGTAGC<br>TCAGGCGGCCGCGAGGGGCAAGGGTGTCTT
**NC-Rab11b**	ATGCACTCGAGCCAGGAAGAGCAGGAGTCC<br>ATCAGGCGGCCGCGAGTTTGGGATGAGGATACAG
**Primers used for qRT-PCR**	
**CD36**	GGCAAAGAACAGCAGCAAAATC<br>TGAAGGCTCAAAGATGGCTCC
**Gck**	CACTGCGGAGATGCTCTTTGAC<br>CCACGATGTTGTTCCCTTCTGC
**Acox2**	GAATAACAGTTGGGGACATAGG<br>CTGGAGGGTGGGTAGGAATC
**Pfkl**	GCAAGGTATGAATGCTGCTGTC<br>TGGAAACGCTGAGCCAGTTGG
**Pctp**	CAAGAAGGGGAGCAGAGAATGG<br>TGGTGTAGCACAGCCAGAGATG
**Rab11b**	CAAAGTGGTGCTTATTGGGGAC<br>CTGAGCCTTGATGGTCTTGCC
**Ces1g**	TGTAAAACCACCACCTCCGCTG<br>TCTCTGGGGTCTCCAAGAAAATC
**Cyp2c29**	ATGTCAAGATTCTGAGCTCCC<br>GTCATTGATAGTGCTTGCCAG
**Tbp**	TATCACTCCTGCCACACCAG<br>CATGATGACTGCAGCAAATCG
	**Oligonucleotides used in Northern blots**
**5s**	TTAGCTTCCGAGATCA
